# First-Trimester Maternal Folic Acid Supplementation Modifies the Effects of Risk Factors Exposures on Congenital Heart Disease in Offspring

**DOI:** 10.3390/life11080724

**Published:** 2021-07-21

**Authors:** Yanji Qu, Shao Lin, Michael S. Bloom, Ximeng Wang, Zhiqiang Nie, Yanqiu Ou, Jinzhuang Mai, Xiangmin Gao, Yong Wu, Jimei Chen, John Justino, Hongzhuan Tan, Jian Zhuang, Xiaoqing Liu

**Affiliations:** 1 Guangdong Cardiovascular Institute, WHO Collaborating Center for Research and Training in Cardiovascular Diseases, Guangdong Provincial People’s Hospital, Guangdong Academy of Medical Sciences, Guangzhou 510080, China; quyanji@gdph.org.cn (Y.Q.); wangximeng@gdph.org.cn (X.W.); niezhiqiang@gdph.org.cn (Z.N.); ouyanqiu@gdph.org.cn (Y.O.); maijinzhuang@gdph.org.cn (J.M.); gaoxiangmin@gdph.org.cn (X.G.); wuyong@gdph.org.cn (Y.W.); chenjimei@gdph.org.cn (J.C.); drzhuangjian5413@163.com (J.Z.); 2 Department of Epidemiology and Health Statistics, Xiangya School of Public Health, Central South University, Changsha 410078, China; tanhz@csu.edu.cn; 3 Department of Environmental Health Sciences, University at Albany, State University of New York, One University Place, Rensselaer, Albany, NY 12144, USA; slin@albany.edu; 4 Department of Epidemiology and Biostatistics, University at Albany, State University of New York, One University Place, Rensselaer, Albany, NY 12144, USA; 5 Department of Global and Community Health, George Mason University, Fairfax, VA 22030, USA; mbloom22@gmu.edu; 6 Department of Health Policy, Management and Behavior, University at Albany, State University of New York, One University Place, Rensselaer, Albany, NY 12144, USA; jjustino@albany.edu

**Keywords:** congenital heart diseases, folic acid, risk factors, interaction

## Abstract

This study aimed to examine effect modification of maternal risk factor exposures and congenital heart disease (CHD) by maternal folic acid supplementation (FAS)/non-FAS. We included 8379 CHD cases and 6918 CHD-free controls from 40 clinical centers in Guangdong Province, Southern China, 2004–2016. Controls were randomly chosen from malformation-free fetuses and infants and frequency matched to the echocardiogram-confirmed cases by enrollment hospital and year of birth. We used multiple regression models to evaluate interactions between FAS/non-FAS and risk factors on CHDs and major CHD categories, adjusted for confounding variables. We detected statistically significant additive and multiplicative interactions between maternal FAS/non-FAS and first-trimester fever, viral infection, and threatened abortion on CHDs. An additive interaction on CHDs was also identified between non-FAS and living in a newly renovated home. We observed a statistically significant dose-response relationship between non-FAS and a greater number of maternal risk factors on CHDs. Non-FAS and maternal risk factors interacted additively on multiple critical CHDs, conotruncal defects, and right ventricular outflow tract obstruction. Maternal risk factor exposures may have differential associations with CHD risk in offspring, according to FAS. These findings may inform the design of targeted interventions to prevent CHDs in highly susceptible population groups.

## 1. Introduction

Congenital heart diseases (CHDs) rank first among birth defects worldwide and affect approximately 1% of all live births [1]. CHDs are also a leading cause of fetal death, infant mortality and morbidity, and long-term disability [2,3]. According to the 2017 Global Burden of Disease Study, more than 11 million individuals live with CHDs globally, and CHDs have caused approximately 89 thousand years lived with a disability [4]. This impact implies a tremendous economic burden for both the affected families and society as a whole. CHDs represent a significant public health issue, but the development of effective primary prevention strategies has been hindered by inadequate understanding of its etiology [5].

The etiology of CHDs is multifactorial, consisting of both genetic and environmental factors. There have been major breakthroughs in understanding the inherited causes of CHDs over the past 50 years, including the identification of specific genetic abnormalities for selected CHD phenotypes [6,7]. However, genetic variations only explain approximately 20% of CHDs [8], while maternal environmental exposures are suspected of contributing to the majority of CHDs. Recognized maternal risk factors for CHDs include: perinatal diseases [9], such as first-trimester viral infection [10], fever [11], hypertension [12], and threatened abortion [13]; maternal reproductive history, including a family history of birth defects [14] and history of abortion (spontaneous and elective) [15]; and lifestyle and environmental factors [9], including alcohol consumption [16], smoking [17], living in a newly renovated home [18], and residing within 50 m of a high traffic roadway [13].

In addition, previous epidemiological studies have also suggested that maternal folic acid supplementation (FAS) was associated with a decreased risk of CHDs in offspring [19,20,21] and may be a feasible intervention. Our recent study found that first-trimester maternal FAS, but not multivitamin use, was associated with a lower risk of CHDs, and the association was strongest for the most severe CHD categories and phenotypes [22]. In China, daily use of FA (0.4 mg), starting from 3 months before to 12 weeks of a planned pregnancy, is generally recommended to prevent neural tube defects (NTDs). The government has provided 0.4 mg FA tablets at no cost since 2009. Although the overall rate of periconceptional FAS has increased significantly since then, more than half of women began FAS after learning of the pregnancy, which frequently missed the critical fetal heart developmental window at 2–8 gestational weeks. In fact, fewer women began FAS before their last menstrual period after the introduction of free-FA in less developed rural areas [23]. In addition, FA food fortification is not mandatory in China. Thus, identifying susceptible women who would most benefit from FAS could provide an opportunity to strengthen the FAS strategy and reduce CHDs.

Although previous studies have assessed individual associations between FAS and other maternal risk factors with CHDs in offspring, the interaction effects of FAS on associations between maternal risk factors and CHDs remains unclear. To help fill this knowledge gap, we examined multiplicative and additive interactions between FAS and CHD risk factors on CHDs. The results of our study will help to design targeted interventions to help prevent CHDs in subpopulations that may benefit most from FAS.

## 2. Materials and Methods

### 2.1. Study Design and Participants

This frequency-matched case-control study was derived from the Guangdong Registry of Congenital Heart Disease (GRCHD), an ongoing CHD registry involving 40 participating centers from 21 cities across Guangdong Province in Southern China [13,24]. All fetuses and newborns delivered at GRCHD centers were actively screened for cardiac anomalies. Fetuses were routinely screened using basic ultrasound at 11–13 gestational weeks (i.e., first trimester) and again at 15–20 gestational weeks (i.e., second trimester). Suspected CHD fetuses were referred for echocardiography to confirm the diagnosis. Genetic tests were performed as needed. All newborns received clinical cardiac assessments before discharge (usually within 72 h of delivery). Suspected CHDs received echocardiographic examination to confirm the diagnosis. Results of other diagnostic procedures, including computed tomography examination, cardiac catheterization, surgery, and autopsy, were also referred to where available. Each CHD case was reviewed, and the diagnosis was confirmed by two senior pediatric cardiologists; disagreements were resolved by a third senior pediatric cardiologist. While CHD diagnoses were coded according to the International Classification of Disease (ICD), 10th Revision (Q20.000–Q28.000), we also applied a unique primary CHD phenotype to each CHD case based on hemodynamics. Only the first record was included for mothers with multiple pregnancies enrolled in the GRCHD. Controls were randomly chosen from singleton fetuses and infants without any congenital malformation and frequency matched to the cases by enrollment hospital and year. CHD cases and controls registered from 2004 to 2016 were eligible.

We focused on isolated CHDs, and excluded CHD cases associated with chromosomal abnormalities, genetic mutations, chromosomal microarray analysis abnormalities, or accompanied by extra-cardiac malformations as recommended by the European Network of Population-based Registries for the Epidemiological Surveillance of Congenital Anomalies (EUROCAT) [25]. CHD cases from multiple gestations were excluded because they might possess different etiology from singletons. We also excluded preterm (<37 weeks gestation at birth) patent ductus arteriosus (PDA) infants, as PDA tends to resolve spontaneously shortly after delivery [26]. Infants older than 1 year of age were excluded to minimize misclassification of self-reported lifestyle factors in the periconceptional period [27].

### 2.2. Data Collection

Parental sociodemographic and lifestyle factors during the periconceptional period were collected by obstetricians via face-to-face interviews, using a structured questionnaire [13]. The questionnaire was administered at the time of enrollment at each participating GRCHD center. As previously described in detail [13], we collected information about maternal sociodemographic factors, first-trimester disease and medication and supplement use, lifestyle, occupational, and environmental exposures during the periconceptional period (i.e., from three months before until the end of the first trimester of pregnancy), reproductive history, and paternal factors during the periconceptional period. We used clinical records to validate self-reported data where feasible.

### 2.3. Classification of CHD Phenotypes

CHD cases were first categorized according to the severity of the primary phenotype as “critical CHDs” if prenatal structural malformations of the heart were present that usually required intervention during the first year of life (mainly including single ventricle, anomalous pulmonary venous return, atrioventricular septal defect, coarctation of aorta, double-outlet right ventricle, hypoplastic left heart syndrome, hypoplastic right heart syndrome, interrupted aortic arch, left ventricular outflow tract obstruction, right ventricular outflow tract obstruction (RVOTO), d-transposition of the great arteries, tetralogy of Fallot, valvular aortic stenosis, and valvular pulmonary stenosis) [28], or as “minor CHDs” (including atrial septal defect and ventricular septal defect). In addition, cases were grouped as “multiple CHDs” if at least two CHD phenotypes were present and “single CHD” if only one CHD phenotype was present. We also combined the classification of CHD phenotype severity and plurality as “multiple critical CHDs”, “single critical CHD”, “multiple minor CHDs”, and “single minor CHD”. Next, to ensure a sufficient sample size for statistical analysis, CHD phenotypes were further categorized according to etiology as conotruncal defects, atrioventricular septal defect (AVSD), anomalous pulmonary venous return (APVR), left ventricle outflow tract obstruction (LVOTO), right ventricle outflow tract obstruction (RVOTO), single ventricle (SV), septal defect, other specified CHD, and unspecified CHD [29].

### 2.4. CHD Risk Factors

We considered maternal risk factors for offspring CHDs as exposures, including first-trimester disease (fever, viral infection, hypertension, and threatened abortion), reproductive history (previous pregnancies with birth defects and spontaneous/elective abortion history), and lifestyle and environmental factors (alcohol consumption and active smoking in the first trimester of pregnancy, living in a newly renovated home (which may increase exposure to air and dust pollutants), and residing within 50 m of a high traffic roadway during periconceptional period, which is associated with greater exposure to air pollutants). We focused on these risk factors as biologically plausible and those for which the associations with CHDs might be modified by FAS. Fever was defined as an axillary body temperature greater than 37 °C. Viral infection was defined as self-reported maternal infection by influenza, hepatitis, rubella, HIV, or herpes virus before or during pregnancy. Hypertension was diagnosed as systolic pressure ≥ 140 mmHg and/or diastolic pressure ≥ 90 mmHg. A threatened abortion was defined as vaginal bleeding before 20 weeks gestational age in conjunction with a positive urine and/or blood pregnancy test and closed cervical os, without passage of products of conception and without evidence of a fetal or embryonic demise [30]. Alcohol consumption referred to self-reported intake of at least 50 mL/day of alcohol drink on average. Active smoking referred to maternal self-reported exposure to at least one cigarette per day on average. Living in a newly renovated home was defined as redecoration and/or remodeling of the maternal residence within 6 months of the periconceptional period.

### 2.5. First-Trimester Maternal FAS

We defined first-trimester maternal “FAS” as reported intake of at least 0.4 mg of FA daily, for more than five days per week, continuously during the first trimester of pregnancy, and anything else as “non-FAS”. The FA tablets were freely distributed by the Chinese government, prescribed, or purchased from other sources. Intake of FA-containing multivitamins was not included as FAS. To facilitate understanding the synergistic effect of risk factors and FAS on CHDs, we used non-FAS as a potential modifier to estimate additive interactions.

### 2.6. Covariates

We considered maternal sociodemographic characteristics and other parental exposures during the periconceptional period that had statistically significant associations (*p* < 0.05) with CHDs as potential confounders, including maternal sociodemographic factors (age, education, household income, residence, and floating population), maternal medication and supplement use during the 1st trimester of pregnancy (traditional Chinese medications and multivitamins), and paternal factors (alcohol consumption and smoking) [13].

### 2.7. Statistical Analysis

We used unconditional logistic regression to estimate associations between FAS and maternal risk factors with CHDs, adjusted for hospital and birth year as matching factors and for the aforementioned covariates as confounders. Exponentiation of the regression coefficients and their corresponding 95% confidence intervals (CIs) provided adjusted odds ratios (OR).

We then conducted stratified analyses to assess potential effect modification by FAS. We tested for multiplicative interactions by including a cross-product term between FAS and each maternal risk factor on CHDs, using Wald’s test to assess statistical significance [31]. We investigated the interaction between risk factor exposures and non-FAS on the additive scale using the relative excess risk due to interaction (RERI) [32]. RERI indicates the extent to which the risk of CHD in the joint presence of both a risk factor and non-FAS (i.e., OR_11_) is greater than the sum of the risks of CHD in the presence of each factor individually (i.e., OR_01_ and OR_10_), such that RERI = OR_11_ − OR_10_ − OR_01_ + 1. We defined a “synergistic” interaction between maternal non-FAS and a maternal risk factor on CHDs as RERI > 0. In addition, we estimated the attributable proportion (AP) and synergy index (S) when RERI > 0 to assess the clinical significance of synergistic interactions. AP reflects the proportion of the joint effect of both non-FAS and a maternal risk factor, that is due to the excess risk, such that AP = RERI/OR_11_. S refers to the ratio between the joint effect and the sum of the individual effects of non-FAS and a risk factor, such that S = (OR_11_ − 1)/((OR_10_ − 1) + (OR_01_ − 1)).

Furthermore, we developed a risk factor index as the sum of exposures to maternal risk factors and estimated the joint effect with non-FAS on CHDs, using FAS mothers without any risk factor exposure as the reference category, and tested for trend. The joint effects of non-FAS with different permutations of individual maternal risk factors on CHDs were also estimated. Finally, we evaluated the additive interaction between non-FAS and synergistic maternal risk factors on the severity, plurality, and etiologic CHD phenotype categories. We used R 3.6.1 (R Core Team, 2019) for all statistical analyses, and statistical significance was defined as *p* < 0.05 for a 2-tailed test.

## 3. Results

We enrolled a total of 15,297 participants in this study, including 8379 CHD cases and 6918 controls without CHDs (Table 1). After adjustment for covariates, first-trimester maternal FAS was associated with a lower risk of CHDs in offspring (OR = 0.69, 95% CI: 0.62–0.76). Conversely, maternal diseases during the first trimester of pregnancy (fever: OR = 2.41, 95% CI: 1.81–3.20; viral infection: OR = 3.00, 95% CI: 2.56–3.52; hypertension: OR = 2.37, 95% CI: 1.52–3.70; and threatened abortion: OR = 1.95, 95% CI: 1.62–2.35), previous pregnancies with birth defects (OR = 6.55, 95% CI: 3.48–12.35), history of spontaneous/elective abortion (OR = 1.28, 95% CI: 1.12–1.47), and maternal lifestyle and environmental factors (living in a newly renovated home: OR = 2.28, 95% CI: 1.74–2.99; and residing within 50 m of a high traffic roadway: OR = 1.16, 95% CI: 1.04–1.30) were associated with increased risks of CHDs in offspring. The frequencies of individual CHD phenotypes are presented in Table S1.

The associations between maternal risk factors and CHDs stratified by FAS and adjusted for covariates are shown in Table 2. The effect estimates of first-trimester maternal fever, viral infection, and threatened abortion and CHDs in offspring were mitigated by first-trimester maternal FAS. We also detected statistically significant multiplicative interactions between maternal FAS and first-trimester fever (*p*-value for interaction = 0.01), viral infection (*p*-value for interaction = 0.01) and threatened abortion (*p*-value for interaction = 0.05).

Table 3 presents the additive interaction effects between non-FAS and risk factors on CHDs. We detected synergistic effects between maternal non-FAS and first-trimester fever (RERI = 2.69, 95% CI: 1.18–4.21), viral infection (RERI = 2.26, 95% CI: 1.22–3.29), and threatened abortion (RERI = 1.23, 95% CI: 0.43–2.03), as well as living in a newly renovated home during the periconceptional period (RERI = 2.51, 95% CI: 1.19–3.84). In addition, the AP was most substantial for non-FAS and living in a newly renovated home (64%), followed by non-FAS and fever (63%), non-FAS and viral infection (48%), and non-FAS and threatened abortion (40%). The S between non-FAS and maternal risk factors was also largest for non-FAS and living in a newly renovated home (7.14, 95% CI: 1.20–42.34). Among the statistically significant additive interactions, we found the greatest risk of CHD among offspring among non-FAS mothers exposed to first-trimester viral infection (OR = 4.73, 95% CI: 3.85–5.80).

**Figure 1:**
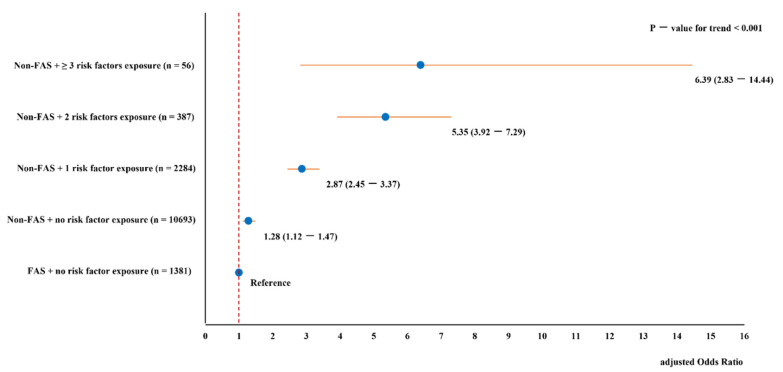
The joint effects of non-folic acid supplementation and exposure to an increasing number of maternal risk factors on congenital heart disease, GRCHD, China, 2004–2016.

The joint effects of non-FAS and exposure to an increasing number of maternal risk factors on CHDs are shown in Figure 1. We found a monotonic increase in CHD risks, although with decreasing precision when non-FAS was coupled with maternal exposure to additional risk factors compared to FAS mothers without co-exposure as the reference group (*p*-value for trend <0.001).

We further estimated the joint effects of non-FAS with exposure to different combinations of individual risk factors on CHDs in offspring, as presented in Table 4. We found that non-FAS mothers with a first-trimester fever and threatened abortion were at the highest risk of CHD in offspring compared to FAS mothers without risk factors exposures (OR = 7.19, 95% CI: 1.58–32.62).

Table S2 shows additive interaction effects between non-FAS and first-trimester viral infection on CHD severity, plurality, and etiology categories. Viral infection had the strongest association with CHD in non-FAS mothers among all maternal risk factors. We found a statistically significant synergistic effect between non-FAS and viral infection for critical CHDs and multiple CHDs, but not for minor CHDs or single CHDs. The joint effect of non-FAS and viral infection was strongest for multiple critical CHDs (OR = 37.63, 95% CI: 24.21–58.47; RERI = 27.45, AP = 0.73, S = 3.99). Among etiologic categories of CHD phenotypes, we detected a statistically significant synergistic effect between non-FAS and viral infection for conotruncal defects (RERI = 23.05, AP = 0.65, S = 3), RVOTO (RERI = 8.75, AP = 0.68, S = 3.77), and other specified CHDs (RERI = 1.54, AP = 0.41, S = 2.26). First-trimester fever threatened abortion, and living in a newly renovated home during the periconceptional period showed synergistic effects with non-FAS on CHD categories similar to viral infection (Tables S3–S5).

## 4. Discussion

### 4.1. Interaction Effects of FAS/Non-FAS, Viral Infection, and Fever on CHDs

We found that FAS significantly mitigated the risk of maternal first-trimester viral infection on CHDs in offspring. In addition, non-FAS and viral infection had synergistic effects on CHDs. The joint effect of non-FAS and viral infection on CHDs was the strongest, in that there were 373% greater odds of CHDs among non-FAS women with a viral infection than for women with non-FAS (50% greater odds) or viral infection (97% greater odds) alone. No previous study investigated the interaction effect of FAS/non-FAS and viral infection on CHDs, so a comparison was impossible. However, maternal infectious disease was previously associated with a greater risk of CHDs in offspring [33]. A meta-analysis involving 17 case-control studies and 67,233 women indicated that early pregnancy maternal viral infection was associated with an approximately two-fold higher odds for CHDs in offspring, which was comparable to our results (OR = 3) [34].

We also found that FAS significantly mitigated the association between maternal first-trimester fever and CHDs in offspring. In addition, the excess risk of non-FAS and fever was the strongest among all risk factors and had the highest RERI value. Similar to our results, previous studies found significant interactions between maternal fever and FAS on CHDs, such that the effect of maternal fever on CHDs among offspring was stronger among non-FAS mothers compared to mothers with FAS [35,36,37]. A previous meta-analysis also found a similar estimate of the association between maternal first-trimester fever and CHDs in offspring (OR = 1.53) to ours (OR = 2.72) [11].

In addition, we found that when viral infection was combined with first-trimester fever, the odds of CHDs were 533% greater among non-FAS mothers than for FAS mothers without exposure to any maternal risk factor. This result implied a synergistic effect of viral infection and fever with non-FAS on CHDs. Similarly, previous evidence suggested greater risks for CHDs associated with influenza infection and fever compared with influenza infection alone [38,39].

Still, the mechanism for an increased risk of CHDs in the offspring of mothers affected by viral infection and fever is unclear, as is the reason for the interaction effect between viral infection, fever, and FAS on CHDs. Cellular apoptosis may be a pathway through which FAS modifies the association between viral infection and fever and CHDs, in that fever and viral infection-induced apoptosis may alter cardiac morphogenesis [40,41,42], while folic acid may rescue folate-deficient apoptotic cells [43]. However, both confirmatory epidemiologic and mechanistic experimental studies will be necessary to more clearly interpret the results.

### 4.2. Synergistic Effects of Non-FAS and Maternal Risk Factors on CHD Categories

We found consistent synergistic effects of non-FAS and maternal risk factors on selected CHDs, including the most complicated multiple critical CHDs and the most common etiological categories of critical CHDs in Asian populations, conotruncal defects, and RVOTO [44]. We found a significant protective association of first-trimester FAS on these CHD phenotypes previously [22]. According to our results, FAS might be recommended for mothers with viral infection during early pregnancy to reduce the risk of conotruncal defects and RVOTO, although a more definitive recommendation will require a randomized trial. Shaw and colleagues previously found a significant interaction between the use of folic acid-containing multivitamins and maternal fever on conotruncal defects [37]. Their case-control study included 207 conotruncal cases and 734 nonmalformed control infants and evaluated the effects of combined maternal vitamin use/fever, no use/no fever, and no use/fever compared to women who reported vitamin use and no periconceptional fever as referents. They found an odds ratio of 2.4 (95% CI: 1.0–5.9) for conotruncal defects when no vitamin use was combined with fever, lower than our estimate of the joint effect of non-FAS and fever on conotruncal defects. Effect differences between FAS and multivitamin use might contribute to the difference between our results and the previous study’s. In our previous work, we did not find similar protective effects of multivitamin use on CHDs as that of FAS [22]. Our results for other detailed etiological categories (e.g., AVSD, APVR, LVOTO, and SV) were unstable due to the limited numbers of cases and so should be considered as preliminary.

### 4.3. Synergistic Effects of Non-FAS and Living in a Newly Renovated Home on CHDs

Living in a newly renovated home was another maternal risk factor with a statistically significant synergistic effect with non-FAS on CHDs. Co-exposure of non-FAS and living in a newly renovated home obtained the highest AP and S among all significant additive effects of non-FAS and risk factors on CHDs. The excess risk of the co-exposures accounted for 64% of the overall risk of CHDs among non-FAS women living in a newly renovated home. The synergistic effect was 7.14 times greater than that of the individual effects of non-FAS and living in a newly renovated home on CHDs. A previous multi-hospital case-control study found that maternal exposure to periconceptional housing renovations was associated with an increased risk of overall CHDs (OR = 1.89, 95% CI: 1.29–2.77), and the association was stronger among first-trimester mothers who moved into a new home within one month of decorating (OR = 4.00, 95% CI: 1.62–9.86), which aligns with our results [18]. However, to the best of our knowledge, ours is the first report of an interaction in which non-FAS potentiated the association between residence in a newly renovated home and CHDs. Thus, the result requires confirmation in another study population using a greater resolution to ascertain materials used during renovation and the duration of exposure at critical developmental windows.

### 4.4. Dose Response and Joint Effects of Non-FAS and Risk Factors Exposures on CHDs

We found that additional risk factors added to the non-FAS association with CHDs in a cumulative manner, suggesting that intervening on behalf of FAS may impact multiple risk scenarios, in which other interventions may be difficult or implausible. In the analysis of the joint effects of non-FAS and multiple CHD risk factors, the combined effect of non-FAS with first-trimester fever and threatened abortion had the strongest association with CHDs in offspring. Similar to our results, threatened abortion was associated with a 1.33-fold higher risk of CHDs in a multi-hospital case-control study from China [13]. Coupled with non-FAS, we found that combined first-trimester fever and threatened abortion were associated with a seven-fold greater risk for CHDs compared to FAS mothers without fever or threatened abortion. These results suggest that the offspring of women with fever and threatened abortion are extremely susceptible to non-FAS for CHD occurrence.

### 4.5. Strengths and Limitations

Although many studies have reported on associations of FAS and other risk factors with CHDs in offspring, this study is novel in being the first, to our knowledge, to quantify interaction effects between FAS/non-FAS and other maternal risk factors on CHDs. The results identified the susceptible group of pregnant women for fetal CHDs who may benefit the most from FAS. Our study has several strengths. The large sample size facilitated our analysis of effect heterogeneity and allowed the investigation of CHD phenotypic subtypes. The geographically diverse study population enrolled across multiple GRCHD sites in Guangdong Province and had a similar CHD prevalence to China overall [44] suggested a representative sample allowing for generalizability of the results. Finally, the CHD diagnoses were confirmed by standardized diagnostic criteria in specialty treatment facilities and validated by specialist physicians, ensuring the validity of the outcome.

However, several limitations should also be considered when interpreting our results. First, recall bias was a concern as we obtained maternal FAS and risk factor information via self-report. We adopted several strategies to minimize recall bias, including: (i) information was collected by obstetricians during face-to-face interviews; (ii) a pregnancy calendar was adopted to help the mothers recall FAS and risk factor exposures; and (iii) detailed information was queried on the type/name, time, and frequency of exposures. Second, selection bias from the enrollment procedure may be a concern because we did not capture all provincial CHD cases. The GRCHD includes 16.5% of tertiary hospitals/maternal and child health hospitals/specialized centers from all geographic areas across the entire province and all CHDs cases occurring in the population being studied. However, the CHD prevalence in the GRCHD was similar to China overall [43], and there was no socioeconomic difference between children delivered in hospitals outside of the GRCHD and those delivered at hospitals in the GRCHD, so the impact was likely to be modest. Third, although we adopted standardized diagnostic rules for diagnosis of CHDs in all GRCHD hospitals/centers, with annual onsite training for participating physicians, and excluded non-isolated CHDs, some cases may have been misclassified. However, outcome misclassification was unlikely to vary by FAS or other maternal risk factors, and so any bias was likely to the null. Fourth, we queried mothers using fairly non-specific risk factor definitions, which may have introduced exposure misclassification. For example, we determined viral infection by self-report, without confirmation from laboratory test reports, and living in a newly renovated home was ascertained by self-report, but not the hazardous components used. A future investigation with a more comprehensive assessment of maternal risk factors is necessary. Fifth, we did not collect information about dietary intake of FA-containing foods, which may confound the association. However, as indicated in our previous work [22], the typical Chinese diet has a lower intake of folate-rich meat than the typical Western diet, and FA-fortified foods are not available in China. In addition, we adjusted the analysis for sociodemographic characteristics, which strongly influence both FAS and diet. Finally, although we identified a large number of overall CHD cases, the limited number of selected CHD phenotypes led to imprecise effect estimates in some strata, and so a larger future study will be necessary to confirm our results.

## 5. Conclusions

We found that first-trimester maternal FAS alleviated the risk of maternal fever, viral infection, and threatened abortion on offspring CHD. There were synergic effects of non-FAS and the aforementioned three risk factors exposures together with living in a newly renovated home on CHDs. There was a significant positive dose-response relationship between non-FAS and CHDs for exposure to an increasing number of risk factors. Based on these observational results, mothers with fever, viral infection, threatened abortion, and living in a newly renovated home might be encouraged to supplement FA early in pregnancy to prevent CHDs. However, results from randomized clinical trials will be necessary to make a more definitive clinical recommendation.

## Figures and Tables

**Table 1:** Maternal first-trimester folic acid supplementation and risk factor exposures for congenital heart disease case and control participants, GRCHD, China, 2004–2016.

Maternal Exposures	Total	CHD Case, n (%)	Control, n (%)	OR (95% CI) *
Total	15297	8379	6918	-
Maternal FAS ^†^				
Yes	1877 (12.3)	928 (11.1)	949 (13.7)	0.69 (0.62-0.76)
No	13,420 (87.7)	7451 (88.9)	5969 (86.3)	1.00 (ref)
Maternal disease ^†^				
Fever				
Yes	371	306 (3.7)	65 (0.9)	2.41 (1.81–3.20)
No	14,926	8073 (96.3)	6853 (99.1)	1.00 (Ref)
Viral infection				
Yes	1114	885 (10.6)	229 (3.3)	3.00 (2.56–3.52)
No	14,183	7494 (89.4)	6689 (96.7)	1.00 (Ref)
Hypertension				
Yes	148	115 (1.4)	33 (0.5)	2.37 (1.52–3.70)
No	15,149	8264 (98.6)	6885 (99.5)	1.00 (Ref)
Threatened abortion				
Yes	727	546 (6.5)	181 (2.6)	1.95 (1.62–2.35)
No	14,567	7831 (93.5)	6736 (97.4)	1.00 (Ref)
Reproductive history				
Previous pregnancies with birth defects				
Yes	106	95 (1.1)	11 (0.2)	6.55 (3.48–12.35)
No	15,191	8284 (98.9)	6907 (99.8)	1.00 (Ref)
Spontaneous/elective abortion history				
Yes	1037	644 (7.7)	393 (5.7)	1.28 (1.12–1.47)
No	14,260	7735 (92.3)	6525 (94.3)	1.00 (Ref)
Maternal lifestyle and environmental factors				
Alcohol consumption ^†^				
Yes	87	65 (0.8)	22 (0.3)	1.65 (0.99–2.73)
No	15,210	8314 (99.2)	6896 (99.7)	1.00 (Ref)
Active Smoking ^†^				
Yes	141	119 (1.4)	22 (0.3)	1.38 (0.82–2.32)
No	15,156	8260 (98.6)	6896 (99.7)	1.00 (Ref)
Living in newly renovated home ^‡^				
Yes	392	317 (3.8)	75 (1.1)	2.28 (1.74–2.99)
No	14,905	8062 (96.2)	6843 (98.9)	1.00 (Ref)
Residing within 50 m of a high traffic roadway ^‡^				
Yes	1721	1076 (12.8)	645 (9.3)	1.16 (1.04–1.30)
No	13,576	7303 (87.2)	6273 (90.7)	1.00 (Ref)

Abbreviations: OR, odds ratio; CHD, congenital heart disease; CI, confidence interval; FAS, folic acid supplementation; GRCHD, Guangdong Registry of Congenital Heart Disease; ref, reference category. * models adjusted for maternal sociodemographic factors (age, education, household income, residence, and floating population), maternal medication/supplement use during the 1st trimester of pregnancy (traditional Chinese medications and multivitamins), and paternal factors (alcohol consumption and smoking); We detected significant collinearities between gravidity and age, previous pregnancies with stillbirths and previous pregnancies with birth defects, a family history of CHDs and previous pregnancies with birth defects. Gravidity, previous pregnancies with stillbirths, and family history were excluded from the multivariable model. ^†^ Exposure window: in the 1st trimester of pregnancy (within 3 months after pregnancy). ^‡^ Exposure window: during the periconceptional period (3 months before pregnancy to the end of the 1st trimester).

**Table 2:** Multiplicative interactions between first-trimester maternal folic acid supplementation and exposure to maternal risk factors on congenital heart disease in offspring, GRCHD, China, 2004–2016.

	With *FAS*		Without *FAS*		
Strata of Maternal Exposures	CHD Cases/ Participants	OR * (95% CI)	CHD Cases/ Participants	OR * (95% CI)	*p*-Value for Multiplicative Interaction
Total	928/1877	-	7451/13,420	-	-
Maternal disease ^†^					
Fever (>38 °C)					
Yes (n = 371)	44/60	1.19 (0.59–2.42)	262/311	2.72 (1.97–3.76)	0.01
No (n = 14,926)	884/1817	1.00 (ref)	7189/13,109	1.00 (ref)	
Viral infection ^‡^					
Yes (n = 1114)	157/218	2.45 (1.73–3.47)	728/896	3.21 (2.68–3.85)	0.01
No (n = 14,183)	771/1659	1.00 (ref)	6723/12,524	1.00 (ref)	
Hypertension					
Yes (n = 148)	12/18	3.17 (0.94–10.69)	103/130	2.34 (1.44–3.82)	0.90
No (n = 15,149)	916/1859	1.00 (ref)	7348/13,290	1.00 (ref)	
Threatened abortion					
Yes (n = 727)	96/152	1.60 (1.08–2.39)	450/575	2.07 (1.67–2.56)	0.05
No (n = 14,570)	832/1725	1.00 (ref)	7001/12,845	1.00 (ref)	
Reproduction history					
Previous pregnancies with birth defects					
Yes (n = 106)	13/15	4.85 (1.01–23.24)	82/91	6.60 (3.28–13.28)	0.61
No (n = 15,191)	915/1862	1.00 (ref)	7369/13,329	1.00 (ref)	
Spontaneous/elective abortion history					
Yes (n = 1037)	123/226	1.10 (0.78–1.54)	521/811	1.23 (1.05–1.45)	0.44
No (n = 14,260)	805/1651	1.00 (ref)	6930/12,609	1.00 (ref)	
Maternal lifestyle and environmental factors					
Alcohol consumption ^†^					
Yes (n = 87)	9/10	5.45 (0.67–44.74)	56/77	1.46 (0.86–2.47)	0.11
No (n = 15,210)	919/1867	1.00 (ref)	7395/13,343	1.00 (ref)	
Active smoking ^†^					
Yes (n = 141)	2/9	1.43 (0.26–7.75)	20/132	1.34 (0.77–2.32)	0.82
No (n = 15,156)	947/1868	1.00 (ref)	5949/13,288	1.00 (ref)	
Living in newly renovated home ^‡^					
Yes (n = 392)	20/30	1.02 (0.42–2.51)	297/360	2.54 (1.91–3.38)	0.93
No (n = 14,905)	908/1847	1.00 (ref)	7154/13,058	1.00 (ref)	
Residing within 50 m of a high traffic roadway ^‡^					
Yes (n = 1721)	94/158	1.29 (0.89–1.89)	982/1563	1.13 (1.00–1.27)	0.93
No (n = 13,576)	834/1719	1.00 (ref)	6469/11,857	1.00 (ref)	

Abbreviations: OR, odds ratio; CHD, congenital heart disease; CI, confidence interval; FAS, folic acid supplementation; GRCHD, Guangdong Registry of Congenital Heart Disease; ref, reference category. * models adjusted for maternal sociodemographic factors (age, education, household income, residence, and floating population), maternal medication/supplement use during the 1st trimester of pregnancy (traditional Chinese medications and multivitamins), and paternal factors (alcohol consumption and smoking); ^†^ Exposure window: in the 1st trimester of pregnancy (within 3 months after pregnancy); ^‡^ Exposure window: during the periconceptional period (3 months before pregnancy to the end of the 1st trimester).

**Table 3:** Additive interactions between maternal FAS/non-FAS and exposure to maternal risk factors on congenital heart disease in offspring, GRCHD, China, 2004–2016 *.

Maternal Exposures	OR_00_ (ref) (FAS, No Exposure)	OR_10_ (95% CI) (Non-FAS, No Exposure)	OR_01_ (95% CI) (FAS, Exposure)	OR_11_ (95% CI) (Non-FAS, Exposure)	RERI (95% CI)	AP (95% CI)	S (95% CI)
Maternal disease ^†^							
Fever	1.00	1.53 (1.37–1.72)	1.08 (0.58–2.00)	4.30 (3.08–6.01)	2.69 (1.18–4.21)	0.63 (0.43–0.82)	5.43 (1.65–17.83)
Viral infection	1.00	1.50 (1.34–1.69)	1.97 (1.42–2.73)	4.73 (3.85–5.80)	2.26 (1.22–3.29)	0.48 (0.32–0.64)	2.53 (1.56–4.10)
Gestational hypertension	1.00	1.56 (1.39–1.75)	1.82 (0.65–5.15)	3.05 (1.92–4.85)	0.67 (−1.67–3.00)	0.22 (−0.49–0.93)	1.48 (0.32–6.79)
Threatened abortion	1.00	1.52 (1.36–1.71)	1.33 (0.92–1.91)	3.08 (2.43–3.90)	1.23 (0.43–2.03)	0.40 (0.20–0.60)	2.45 (1.27–4.73)
Reproductive history							
Previous pregnancy with birth defect	1.00	1.40 (1.23–1.58)	4.49 (0.96–21.05)	9.78 (4.83–19.82)	4.90 (−4.83–14.63)	0.00 (−0.29–1.29)	2.26 (0.32–15.8)
Spontaneous/elective abortion history	1.00	1.39 (1.22–1.57)	1.17 (0.86–1.58)	1.80 (1.49–2.18)	0.24 (−0.20–0.69)	0.14 (−0.10–0.37)	1.44 (0.69–3.00)
Maternal lifestyle and environmental factors							
Alcohol consumption ^†^	1.00	1.57 (1.40–1.76)	8.23 (1.02–66.39)	2.28 (1.34–3.90)	−6.51 (−23.72–10.7)	−2.85 (−10.63–4.92)	0.16 (0.02–1.80)
Active smoking ^†^	1.00	1.40 (1.24–1.59)	1.23 (0.23–6.66)	1.77 (1.01–3.09)	1.43 (−2.17–5.02)	0.47 (0.09–0.84)	3.25 (1.07–9.86)
Living in newly renovated home ^‡^	1.00	1.54 (1.38–1.73)	0.87 (0.39–1.93)	3.92 (2.89–5.32)	2.51 (1.19–3.84)	0.64 (0.44–0.84)	7.14 (1.20–42.34)
Residing within 50 m of a high traffic roadway ^‡^	1.00	1.56 (1.39–1.75)	1.07 (0.75–1.54)	1.70 (1.46–1.99)	0.07 (−0.36–0.50)	0.04 (−0.21–0.29)	1.11 (0.57–2.17)

Abbreviations: AP, attributable proportion; CHD, congenital heart disease; CI, confidence interval; FAS, folic acid supplementation; GRCHD, Guangdong Registry of Congenital Heart Disease; OR, odds ratio; RERI, relative excess risk due to interaction; S, synergy index. * models adjusted for maternal sociodemographic factors (age, education, household income, residence, and floating population), maternal medication/supplement use during the 1st trimester of pregnancy (traditional Chinese medications and multivitamins), and paternal factors (alcohol consumption and smoking); ^†^ Exposure window: in the 1st trimester of pregnancy (within 3 months after pregnancy); ^‡^ Exposure window: during the periconceptional period (3 months before pregnancy to the end of the 1st trimester).

**Table 4:** Joint effects of maternal non-folic acid supplementation and exposure to risk factors on congenital heart disease in offspring, GRCHD, China, 2004–2016.

Exposures	n	OR (95% CI) *
FAS + No risk factor exposure	1381	1.00 (Reference)
Non-FAS + Viral infection + Living in newly renovated home	30	4.45 (1.74–11.40)
Non-FAS + Viral infection + Threatened abortion	42	4.87 (2.17–10.92)
Non-FAS + Fever + Living in newly renovated home	6	5.63 (0.61–52.35)
Non-FAS + Threatened abortion + Living in newly renovated home	94	6.19 (2.77–13.86)
Non-FAS + Fever + Viral infection	92	6.33 (3.47–11.56)
Non-FAS + Fever + Threatened abortion	17	7.19 (1.58–32.62)

Abbreviations: OR, odds ratio; CI, confidence interval; FAS, folic acid supplementation; GRCHD, Guangdong Registry of Congenital Heart Disease. * models adjusted for maternal sociodemographic factors (age, education, household income, residence, and floating population), maternal medication/supplement use during the 1^st^ trimester of pregnancy (traditional Chinese medications and multivitamins), and paternal factors (alcohol consumption and smoking).

## Data Availability

Data are available upon reasonable request.

## Supplementary Materials

The following are available online at https://www.mdpi.com/article/10.3390/life11080724/s1, Table S1: Number and frequencies of congenital heart diseases categories and phenotypes, GRCHD study, 2004–2016; Table S2: Additive interaction between maternal non-folic acid supplementation and first-trimester maternal fever on congenital heart disease categories, GRCHD, China, 2004–2016; Table S3: Additive interaction between maternal non-folic acid supplementation and first trimester threatened abortion on congenital heart disease categories, GRCHD, China, 2004–2016; Table S4: Additive interaction between maternal non-folic acid supplementation and living in a newly renovated home during the periconceptional period on congenital heart disease categories, GRCHD, China, 2004–2016.
