# Supplementary material for: First-Trimester Maternal Folic Acid Supplementation Modifies the Effects of Risk Factors Exposures on Congenital Heart Disease in Offspring
Source: Life (Basel). 2021 Jul 21;11(8):724. doi: 10.3390/life11080724 (PMC8399198; doi:10.3390/life11080724)
Supplement: Supplementary file 1 [file life-11-00724-s001.zip › life-1243518-supplementary.pdf]

Table S1. Frequencies of congenital heart diseases, by categories and phenotypes, GRCHD, 2004-2016.

CHD phenotypes	N	%
Total CHDs	8379	100.00
Conotruncal defects	868	10.36
TGA	343	4.09
ToF	309	3.69
DORV	178	2.12
Truncus arteriosus	38	0.45
AVSD	184	2.20
APVR	77	0.92
LVOTO	213	2.54
CoA/IAA	111	1.32
HLHS	61	0.73
vAS	41	0.49
RVOTO	536	6.40
HRHS	74	0.88
Ebstein anomaly	43	0.51
PA	68	0.81
vPS	351	4.19
SV	144	1.72
Septal defects	4437	52.95
VSD	2863	34.17
ASD	1574	18.79
Other specified CHDs	1385	16.53
Unspecified CHDs	535	6.39

Abbreviations: ASD, atrial septal defect; APVR, anomalous pulmonary venous return; AVSD, atrioventricular septal defect; CHD, congenital heart disease; CoA, coarctation of aorta; DORV, double outlet right ventricle; HLHS, hypoplastic left heart syndrome; HRHS, hypoplastic right heart syndrome; IAA, interrupted aortic arch; LVOTO, left ventricle outflow tract obstruction; PA, pulmonary atresia; RVOTO, right ventricle outflow tract obstruction; SV, single ventricle; TGA, d-transposition of the great arteries; ToF, tetralogy of Fallot; vAS, valvular aortic stenosis; vPS, valvular pulmonary stenosis; VSD, ventricular septal defect.

Table S2. Additive interactions between maternal FAS/non-FAS and first trimester maternal viral infection on congenital heart disease categories, GRCHD, China, 2004-2016 [*, †].

CHD categories	N	OR_{11} (95%CI) (non-FAS=1 & viral infection=1)	RERI (95%CI)	AP (95%CI)	S (95% CI)
Severity categories					
Critical CHDs	3906	8.18 (6.37-10.50)	3.59 (1.47-5.72)	0.44 (0.24-0.63)	2 (1.30-3.08)
Minor CHDs	4473	2.97 (2.33-3.77)	0.88 (-0.04-1.8)	0.3 (0.02-0.57)	1.81 (0.88-3.76)
Plurality categories					
Multiple CHDs	2879	10.75 (8.11-14.26)	6.79 (4.00-9.57)	0.63 (0.49-0.77)	3.29 (2.02-5.36)
Single CHDs	5500	3.21 (2.55-4.03)	0.46 (-0.59-1.50)	0.14 (-0.17-0.45)	1.26 (0.72-2.20)
Severity and plurality categories					
Multiple Critical CHDs	1572	37.63 (24.21-58.47)	27.45 (13.64-41.27)	0.73 (0.62-0.84)	3.99 (2.53-6.3)
Single Critical CHDs	2334	3.31 (2.47-4.44)	-0.28 (-1.76-1.19)	-0.09 (-0.54-0.37)	0.89 (0.49-1.6)
Multiple Minor CHDs	1307	2.86 (1.98-4.14)	0.81 (-0.51-2.12)	0.28 (-0.13-0.70)	1.77 (0.59-5.29)
Single Minor CHDs	3166	2.88 (2.23-3.73)	0.77 (-0.22-1.76)	0.27 (-0.05-0.58)	1.69 (0.77-3.70)
Etiologic categories					
Conotruncal defects	868	35.57 (19.44-65.09)	23.05 (6.42-39.69)	0.65 (0.46-0.83)	3.00 (1.71-5.27)
AVSD	184	24.45 (7.74-77.28)	16.95 (-5.43-39.34)	0.69 (0.32-1.06)	3.61 (0.91-14.38)
APVR	77	7.03 (2.10-23.59)	6.50 (-0.86-13.86)	0.92 (0.76-1.08)	-13.20 (NA)
LVOTO	213	23.70 (9.72-57.75)	13.69 (-3.80-31.17)	0.58 (0.19-0.97)	2.52 (0.91-6.94)
RVOTO	536	12.91 (7.62-21.87)	8.75 (2.80-14.71)	0.68 (0.44-0.92)	3.77 (1.43-9.92)
SV	144	157.12 (21.26-1161.32)	NA	0.77 (0.48-1.06)	4.35 (1.21-15.68)
Septal defects	4437	2.94 (2.32-3.74)	0.88 (-0.03-1.80)	0.30 (0.02-0.58)	1.84 (0.88-3.85)
Other specified CHDs	1385	3.76 (2.63-5.37)	1.54 (0.10-2.97)	0.41 (0.10-0.72)	2.26 (0.90-5.63)

Abbreviations: AP, attributable proportion; APVR, anomalous pulmonary venous return; AVSD, atrioventricular septal defect; CHD, congenital heart diseases; CI, confidence intervals; FAS, folic acid supplementation; GRCHD, Guangdong Registry of Congenital Heart Disease; LVOTO, left ventricular outflow tract obstruction; NA, not available; OR, odds ratio; RERI, relative excess risk due to interaction; RVOTO, right ventricular outflow tract obstruction; S, synergy index; SV, single ventricle;

[*] models adjusted for maternal sociodemographic factors (age, education, household income, residence, and floating population), maternal medication/supplement use during the 1st trimester of pregnancy (traditional Chinese medications and multivitamins), and paternal factors (alcohol consumption and smoking);

[†] 535 unspecified CHDs excluded.

Table S3. Additive interactions between maternal FAS/non-FAS and first trimester maternal fever on congenital heart disease categories, GRCHD, China, 2004-2016 *, †

CHD categories	OR_{00} (ref) (FAS, no exposure)	OR_{10} (95%CI) (Non-FAS, no exposure)	OR_{01} (95%CI) (FAS, exposure)	OR_{11} (95%CI) (Non-FAS, exposure)	RERI (95%CI)
Severity categories					
Critical CHDs	1.00 (ref)	1.73 (1.47-2.04)	2.55 (1.24-5.22)	6.74 (4.63-9.82)	3.46 (0.48-6.44)
Minor CHDs	1.00 (ref)	1.07 (0.93-1.22)	1.93 (0.98-3.79)	4.30 (3.00-6.17)	2.31 (0.36-4.26)
Plurality categories					
Multiple CHDs	1.00 (ref)	2.20 (1.80-2.70)	2.68 (1.12-6.39)	9.05 (6.03-13.59)	5.16 (1.09-9.24)
Single CHDs	1.00 (ref)	1.08 (0.95-1.23)	2.01 (1.07-3.78)	4.16 (2.93-5.91)	2.07 (0.2-3.94)
Severity and plurality categories					
Multiple Critical CHDs	1.00 (ref)	4.77 (3.34-6.81)	2.21 (0.48-10.14)	19.83 (11.66-33.73)	13.85 (4.02-23.68)
Single Critical CHDs	1.00 (ref)	1.17 (0.98-1.40)	2.41 (1.14-5.10)	4.18 (2.74-6.37)	1.6 (-0.82-4.02)
Multiple Minor CHDs	1.00 (ref)	1.25 (0.99-1.59)	2.66 (1.02-6.97)	4.81 (2.94-7.88)	1.89 (-1.41-5.2)
Single Minor CHDs	1.00 (ref)	0.99 (0.85-1.15)	1.65 (0.79-3.43)	4.08 (2.78-5.98)	2.44 (0.55-4.34)
Etiologic categories					
Conotruncal defects	1.00 (ref)	4.69 (2.92-7.55)	1.93 (0.24-15.72)	26.71 (14.06-50.72)	21.08 (5.37-36.79)
AVSD	1.00 (ref)	4.06 (1.53-10.74)	NA	15.58 (4.28-56.70)	12.52 (-4.9-29.94)
APVR	1.00 (ref)	1.69 (0.62-4.64)	NA	9.98 (2.38-41.90)	9.29 (-3.96-22.53)
LVOTO	1.00 (ref)	2.86 (1.41-5.83)	NA	5.90 (1.70-20.54)	4.04 (-2.43-10.51)
RVOTO	1.00 (ref)	2.29 (1.48-3.55)	5.62 (1.48-21.32)	12.08 (6.20-23.52)	5.17 (-4.7-15.04)
SV	1.00 (ref)	NA	NA	NA	NA
Septal defects	1.00 (ref)	1.06 (0.92-1.22)	1.85 (0.94-3.66)	4.31 (3.01-6.19)	2.4 (0.47-4.33)
Other specified CHDs	1.00 (ref)	1.47 (1.16-1.86)	2.19 (0.82-5.83)	2.81 (1.58-4.98)	0.15 (-2.43-2.73)

Abbreviations: APVR, anomalous pulmonary venous return; AVSD, atrioventricular septal defect; CHD, congenital heart diseases; CI, confidence intervals; FAS, folic acid supplementation; GRCHD, Guangdong Registry of Congenital Heart Disease; LVOTO, left ventricular outflow tract obstruction; NA, not available; OR, odds ratio; RERI, relative excess risk due to interaction; RVOTO, right ventricular outflow tract obstruction; SV, single ventricle;

* models adjusted for maternal sociodemographic factors (age, education, household income, residence, and floating population), maternal medication/supplement use during the 1st trimester of pregnancy (traditional Chinese medications and multivitamins), and paternal factors (alcohol consumption and smoking);

† 535 unspecified CHDs excluded.

Table S4. Additive interactions between maternal FAS/non-FAS and first trimester threatened abortion on congenital heart disease categories, GRCHD, China, 2004-2016 *, †

CHD categories	OR_{00} (ref) (FAS, no exposure)	OR_{10} (95%CI) (Non-FAS, no exposure)	OR_{01} (95%CI) (FAS, exposure)	OR_{11} (95%CI) (Non-FAS, exposure)	RERI (95%CI)
Severity categories					
Critical CHDs	1.00 (ref)	1.75 (1.48-2.06)	2.28 (1.46-3.54)	5.02 (3.81-6.62)	2 (0.4-3.53)
Minor CHDs	1.00 (ref)	1.06 (0.92-1.22)	1.39 (0.91-2.14)	2.26 (1.73-2.95)	0.81 (0.02-1.59)
Plurality categories					
Multiple CHDs	1.00 (ref)	2.16 (1.76-2.66)	1.70 (0.92-3.14)	5.88 (4.29-8.05)	3.02 (1.18-4.86)
Single CHDs	1.00 (ref)	1.09 (0.95-1.24)	1.69 (1.15-2.48)	2.46 (1.91-3.16)	0.68 (-0.15-1.52)
everity and plurality categories					
Multiple Critical CHDs	1.00 (ref)	4.89 (3.39-7.06)	2.68 (1.08-6.68)	15.25 (9.68-24.03)	8.67 (2.93-14.41)
Single Critical CHDs	1.00 (ref)	1.18 (0.98-1.41)	2.18 (1.37-3.46)	3.03 (2.22-4.13)	0.67 (-0.58-1.93)
Multiple Minor CHDs	1.00 (ref)	1.22 (0.96-1.54)	1.27 (0.59-2.72)	2.65 (1.79-3.94)	1.17 (-0.12-2.45)
Single Minor CHDs	1.00 (ref)	1.00 (0.86-1.17)	1.43 (0.91-2.26)	2.09 (1.57-2.78)	0.65 (-0.17-1.47)
Etiologic categories					
Conotruncal defects	1.00 (ref)	5.14 (3.12-8.46)	3.41 (1.11-10.47)	13.72 (7.51-25.06)	6.17 (-0.51-12.86)
AVSD	1.00 (ref)	6.16 (1.86-20.37)	13.16 (2.11-82.13)	20.28 (5.39-76.34)	1.96 (-21.39-25.31)
APVR	1.00 (ref)	1.65 (0.60-4.48)	NA	4.33 (1.11-16.94)	3.69 (-1.24-8.62)
LVOTO	1.00 (ref)	2.99 (1.44-6.24)	3.39 (0.72-15.92)	10.86 (4.37-26.99)	5.48 (-3.52-14.47)
RVOTO	1.00 (ref)	2.05 (1.33-3.14)	0.62 (0.08-4.70)	6.93 (3.89-12.36)	5.27 (1.69-8.84)
SV	1.00 (ref)	12.61 (1.75-90.73)	NA	32.40 (3.96-264.94)	20.79 (-25.02-66.6)
Septal defects	1.00 (ref)	1.06 (0.92-1.22)	1.40 (0.91-2.15)	2.32 (1.78-3.03)	0.86 (0.07-1.66)
Other specified CHDs	1.00 (ref)	1.37 (1.08-1.74)	0.44 (0.16-1.26)	2.52 (1.69-3.74)	1.7 (0.74-2.67)

Abbreviations: APVR, anomalous pulmonary venous return; AVSD, atrioventricular septal defect; CHD, congenital heart diseases; CI, confidence intervals; FAS, folic acid supplementation; GRCHD, Guangdong Registry of Congenital Heart Disease; LVOTO, left ventricular outflow tract obstruction; NA, not available; OR, odds ratio; RERI, relative excess risk due to interaction; RVOTO, right ventricular outflow tract obstruction; SV, single ventricle;

* models adjusted for maternal sociodemographic factors (age, education, household income, residence, and floating population), maternal medication/supplement use during the 1st trimester of pregnancy (traditional Chinese medications and multivitamins), and paternal factors (alcohol consumption and smoking);

† 535 unspecified CHDs excluded.

Table S5. Additive interactions between maternal FAS/non-FAS and living in newly renovated home during periconceptional period on congenital heart disease categories, GRCHD, China, 2004-2016 *,†

CHD categories	OR_{00} (ref) (FAS, no exposure)	OR_{10} (95%CI) (Non-FAS, no exposure)	OR_{01} (95%CI) (FAS, exposure)	OR_{11} (95%CI) (Non-FAS, exposure)	RERI (95%CI)
Severity categories					
Critical CHDs	1.00 (ref)	1.72 (1.46-2.03)	3.13 (1.32-7.40)	6.19 (4.39-8.72)	2.34 (-0.92-5.61)
Minor CHDs	1.00 (ref)	1.05 (0.92-1.21)	0.62 (0.21-1.81)	2.60 (1.86-3.64)	1.93 (0.88-2.98)
Plurality categories					
Multiple CHDs	1.00 (ref)	2.20 (1.79-2.69)	2.42 (0.82-7.11)	6.47 (4.38-9.54)	2.85 (-0.54-6.25)
Single CHDs	1.00 (ref)	1.07 (0.94-1.21)	1.21 (0.51-2.87)	3.05 (2.22-4.18)	1.77 (0.4-3.14)
Severity and plurality categories					
Multiple Critical CHDs	1.00 (ref)	4.85 (3.39-6.94)	2.81 (0.56-14.08)	13.90 (8.13-23.77)	7.24 (-0.32-14.79)
Single Critical CHDs	1.00 (ref)	1.15 (0.96-1.37)	2.89 (1.18-7.08)	4.38 (3.03-6.33)	1.34 (-1.59-4.27)
Multiple Minor CHDs	1.00 (ref)	1.23 (0.97-1.56)	1.89 (0.54-6.63)	3.72 (2.34-5.94)	1.6 (-1.19-4.4)
Single Minor CHDs	1.00 (ref)	0.98 (0.84-1.14)	0.20 (0.04-0.98)	2.19 (1.52-3.16)	2.01 (1.19-2.82)
Etiologic categories					
Conotruncal defects	1.00 (ref)	4.64 (2.90-7.43)	NA	12.25 (6.22-24.10)	8.6 (1.61-15.59)
AVSD	1.00 (ref)	5.11 (1.75-14.92)	20.49 (1.90-221.03)	20.92 (5.46-80.16)	-3.68 (-51.68-44.31)
APVR	1.00 (ref)	1.75 (0.64-4.78)	NA	5.68 (1.16-27.92)	4.93 (-3.17-13.04)
LVOTO	1.00 (ref)	2.80 (1.37-5.70)	NA	15.40 (5.70-41.56)	13.6 (-0.38-27.58)
RVOTO	1.00 (ref)	2.22 (1.44-3.40)	2.19 (0.25-19.03)	5.23 (2.42-11.33)	1.83 (-3.95-7.61)
SV	1.00 (ref)	13.53 (1.88-97.36)	NA	39.78 (4.43-357.16)	27.25 (-37.31-91.81)
Septal defects	1.00 (ref)	1.05 (0.92-1.21)	0.62 (0.21-1.81)	2.60 (1.85-3.63)	1.92 (0.87-2.97)
Other specified CHDs	1.00 (ref)	1.42 (1.12-1.80)	1.38 (0.36-5.32)	4.54 (2.90-7.11)	2.75 (0.17-5.32)

Abbreviations: APVR, anomalous pulmonary venous return; AVSD, atrioventricular septal defect; CHD, congenital heart diseases; CI, confidence intervals; FAS, folic acid supplementation; GRCHD, Guangdong Registry of Congenital Heart Disease; LVOTO, left ventricular outflow tract obstruction; NA, not available; OR, odds ratio; RERI, relative excess risk due to interaction; RVOTO, right ventricular outflow tract obstruction; SV, single ventricle;

* models adjusted for maternal sociodemographic factors (age, education, household income, residence, and floating population), maternal medication/supplement use during the 1st trimester of pregnancy (traditional Chinese medications and multivitamins), and paternal factors (alcohol consumption and smoking);

† 535 unspecified CHDs excluded.
